# Veterinary and human vaccine evaluation methods

**DOI:** 10.1098/rspb.2013.2839

**Published:** 2014-06-07

**Authors:** T. J. D. Knight-Jones, K. Edmond, S. Gubbins, D. J. Paton

**Affiliations:** 1 The Pirbright Institute, Pirbright, UK; 2 The Royal Veterinary College (VEEPH), University of London, London, UK; 3 School of Paediatrics and Child Health (SPACH), The University of Western Australia, Crawley, Australia

**Keywords:** vaccine, evaluation, veterinary, vaccine effectiveness

## Abstract

Despite the universal importance of vaccines, approaches to human and veterinary vaccine evaluation differ markedly. For human vaccines, *vaccine efficacy* is the proportion of vaccinated individuals protected by the vaccine against a defined outcome under ideal conditions, whereas for veterinary vaccines the term is used for a range of measures of vaccine protection. The evaluation of *vaccine effectiveness*, vaccine protection assessed under routine programme conditions, is largely limited to human vaccines. Challenge studies under controlled conditions and sero-conversion studies are widely used when evaluating veterinary vaccines, whereas human vaccines are generally evaluated in terms of protection against natural challenge assessed in trials or post-marketing observational studies. Although challenge studies provide a standardized platform on which to compare different vaccines, they do not capture the variation that occurs under field conditions. Field studies of vaccine effectiveness are needed to assess the performance of a vaccination programme. However, if vaccination is performed without central co-ordination, as is often the case for veterinary vaccines, evaluation will be limited. This paper reviews approaches to veterinary vaccine evaluation in comparison to evaluation methods used for human vaccines. Foot-and-mouth disease has been used to illustrate the veterinary approach. Recommendations are made for standardization of terminology and for rigorous evaluation of veterinary vaccines.

## 1. Introduction

Vaccines are crucial in the control of many human and veterinary diseases. Routine vaccination is used by most countries in the world to control about 15–20 human infectious diseases, and roughly another 15 diseases are selectively targeted [1]. It is estimated that veterinary vaccines are available for over 400 diseases affecting mammals, birds and fish, including farm animals, pets and wildlife [2]. Though revenues from the global human vaccine market are over 30 times that of veterinary vaccines [2], veterinary vaccines are very widely used with over two billion doses of foot-and-mouth disease (FMD) vaccine used per year [3], and poultry vaccines given on an even greater scale [4].

Given the enormous scale and implications of vaccine use in terms of both health and economics, it is clearly important that their effectiveness be thoroughly evaluated. In fact, human and veterinary vaccines are evaluated in very different ways. Here, we review the various approaches to vaccine evaluation and discuss both the rationale for these different approaches and the problems encountered.

### (a) Fundamental differences

Despite the large number of veterinary vaccines in use, the literature on their evaluation is small compared with that in the human vaccine field; this is exacerbated by a failure to publish findings by vaccine manufacturers***.*** A basic terminology exists employing the words *efficacy*, *effectiveness* and *coverage*; yet, these terms are inconsistently used in the veterinary world (table 1). This is partially explained by the range of disease outcomes targeted by livestock vaccines to improve profitability (see examples in table 2).

**Table 1.** Definition and usage of terms in human and veterinary vaccine evaluation.

term		definition
vaccine potency	veterinary	‘relative strength of a biological product as determined by appropriate test methods. (Initially the potency is measured using an efficacy test in animals*… with pathogen challenge;*… later this may be correlated with tests of antigen content, or antibody response, for routine batch potency tests.)’ [5]
	human	‘potency is the specific ability or capacity of the vaccine as measured by a laboratory test’ [6]
	*difference*	*similar definition but less frequently used for human vaccine evaluation*
vaccine efficacy	veterinary	‘specific ability of the biological product to produce the result for which it is offered when used under the conditions recommended by the manufacturer’ [5]
		‘the ability of the vaccine to give protection against the adverse effects of the infection to the vaccinated animal...’ [7]
	human	‘...the percentage reduction in disease incidence attributable to vaccination [usually] calculated by means of the following equation: 1.1 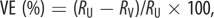 where *R*_U_ = the incidence risk or rate in unvaccinated people and *R*_V_ = the incidence in vaccinated people … measured in an individually randomized placebo-controlled clinical trial’ [1]. The equation for vaccine efficacy can be reformulated as: 1.2 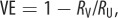 where *R*_V_*/*R_U_ is the relative risk or rate ratio.
	*difference*	*veterinary usage has not been standardized*
vaccine effectiveness	veterinary	usually not a specific term, more the ability of a vaccine to control disease in the field [8]
	human	vaccine efficacy measured by observational studies under field conditions within a vaccination programme [1] or measured by trials conducted under normal programme conditions
correlate of protection	veterinary	a variety of terms are used to describe this widely used concept
	human	a specific response to a vaccine that is associated with protection against infection, disease, or other defined endpoint [9,10]
vaccine coverage	veterinary	as for human—although occasionally it refers to the proportion of the target population that have sero-converted to a protective titre; the latter is sometimes called immunization coverage or population immunity [11]
	human	the proportion of the target population that have been vaccinated according to a defined schedule. Sometimes called immunization coverage [12]
	*difference*	*occasionally in veterinary programmes ‘immunization coverage’ may refer to the proportion that have sero-converted above a titre deemed protective*

**Table 2.** Examples of disease outcomes targeted by veterinary vaccines, other than clinical disease in vaccinated animals.

outcome	disease examples
mortality	clostridial diseases, rinderpest, cattle lungworm
abortion rate	porcine parvovirus and porcine reproductive and respiratory syndrome (PRRS). Infectious bovine rhinotracheitis, bovine viral diarrhoea (BVD) and salmonellosis in cattle. *Chlamydophila abortus* and toxoplasmosis in sheep. Equine herpesvirus
weight gain and efficiency of feed conversion into meat	PRRS, porcine circovirus
disease transmission rates	various including porcine circovirus and FMD
shedding of zoonotic pathogens (to protect human health)	*Salmonella enteritidis* in hen eggs, *Escherichia coli* O157 in cattle
morbidity in offspring after vaccination of dams	various including rotavirus and *E. coli* in cattle
protection against fetal infection *in utero*	BVD

Although herd effects may be considered [13], the outcome of interest for human vaccine evaluations is typically the status of the individual. In veterinary medicine, assessment of overall group status is common, as management is often done at the group level. This results in the lack of individual data and analysis. This extends to disease control, where spread between herds may be of greater concern than spread within already infected herds. However, there are potential problems with evaluation at the group level, including a failure to account for population turnover and variation in immunity and pathogen exposure within a group. These inaccuracies and confounders can lead to a limited or incorrect understanding of vaccine protection.

For certain notifiable animal diseases, zonal or national disease-free status is required to gain access to lucrative international export markets for animals and their products. This leads to a focus on regional pathogen eradication. For human disease control, the emphasis is on reducing morbidity regardless of infection status. Exceptions include elimination programmes (e.g. polio) and novel malaria vaccines that block transmission. Options for restricting contact between infected and susceptible humans are limited, depending instead on immunity to control disease. This differs from the control of important veterinary diseases where the use of culling and movement controls is long-established. Furthermore, immunization may be prohibited where disease-free status must be proved and it is not possible to distinguish vaccinated and infected animals.

As livestock are ultimately economic commodities, besides welfare considerations, disease control must be profitable. This limits resources available for veterinary vaccine development and application. Human vaccines may cost more than US$100 per dose, by contrast an individual chicken is worth only a few dollars. Although higher prices may be paid for pets and breeding stock, the size of the market is small.

### (b) Stages of evaluation

A vaccine may be evaluated during development, licensing and introduction, vaccine batch testing, programme monitoring or after suspected vaccine failure. To obtain licensure, human vaccines typically undergo initial evaluation in animal models followed by a series of controlled trials (phase I, II and III; electronic supplementary material, table S1) with an increasing number of human subjects to assess safety, immunogenicity and then *efficacy* (defined in table 1) [1]. National health ministries then evaluate such information before allowing vaccines to be introduced. If a vaccine is to be used in a state-funded programme, cost-effectiveness will also be evaluated. After licensure and introduction of a human vaccine, protection in the field against natural challenge is estimated by means of observational studies and called *vaccine effectiveness* (phase IV) [12].

In order for veterinary vaccines to obtain market authorization, they are subjected to safety and immunogenicity studies on a limited number of individuals of the target species [14–17]. Their ability to protect is assessed by *in vivo* challenge or occasionally by sero-conversion studies, the results of which have been expressed using a variety of different statistics often called measures of *efficacy* [5,14]. Although they are used in the assessment of efficacy, the scale of veterinary vaccine field studies are limited compared with human vaccine trials.

Only on rare occasions are field studies not required at all for licensure of a veterinary vaccine in the European Union, e.g. when pathogen challenge in the field is unreliable or when rapid licensure is required during an emergency situation. Field trials may not be possible when vaccination is prohibited, as is often the case for exotic notifiable animal diseases in countries needing to prove free status [14,16,17]. However, in less regulated parts of the world, field studies play a very limited role in veterinary vaccine authorization and are typically used to evaluate safety rather than efficacy [5].

## 2. Evaluating protective effects in vaccinated humans and animals

Below, we consider a hierarchy of studies employed for the evaluation of human and veterinary vaccines (summarized in table 3). These different designs vary in both the value of evidence that they provide and their resource requirements.

**Table 3.** The differing evaluation methods for human and veterinary vaccines.

evaluation method	human usage	veterinary usage
challenge studies	initial evaluation with animal models subsequent human challenge studies are performed for certain pathogens	initial and final vaccine efficacy testing
randomized trials	individual and cluster randomized trials routinely used for licensure efficacy evaluation sometimes for post-licensure effectiveness evaluation	usually used for licensure efficacy evaluation
post-vaccination immune correlate response	often used pre-licensure and occasionally for licensure	often used pre and post-licensure
vaccine effectiveness observational field studies	routinely used for monitoring post-licensure	rarely performed
vaccine effectiveness observational studies using routine surveillance data	routinely used for monitoring post-licensure when adequate data are available	not performed
post-vaccination sero-conversion field surveys	rarely used	often used for monitoring post-licensure
sero-prevalence population immunity surveys	rarely used	often used for monitoring post-licensure
*in vitro* serological matching assays	used post-licensure when suitable assay exists	often used post-licensure when suitable assay exists
coverage evaluation	various methods routinely used	distributed method sometimes used

### (a) Challenge studies

Vaccinated and unvaccinated individuals may be compared after direct challenge with the target pathogen under controlled experimental conditions. Challenging humans with dangerous pathogens is rarely acceptable. However, challenge studies using animal models are important for the initial evaluation of human vaccines. Challenge studies with human subjects are sometimes performed for pathogens with effective treatments, such as specific strains of malaria, and where the disease is usually self-limiting, such as typhoid, cholera, the common cold and influenza [18]. Human challenge is also used to assess protection against an attenuated pathogen, for example oral polio vaccine [19].

The evaluation of veterinary vaccines relies heavily on challenge studies. Typically, protection is assessed using a high level of pathogen challenge with the lowest vaccine antigen content permitted under the authorization. Although this will provide some confidence that the vaccine will protect even in extreme situations, the controlled conditions of a challenge study will not reflect the sometimes suboptimal application of vaccines in the field. For some important veterinary pathogens, the design of these challenge studies is prescribed by official standards (box 1) [5,16].

Box 1. **FMD 50% protective dose (PD_50_).**

In Europe, FMD vaccines are routinely evaluated using the PD_50_ test.

Three groups of at least five cattle are given different doses of vaccine (typically a full, a quarter and a 16th dose). Two unvaccinated control animals are also used. After three to four weeks, animals are given a standard dose of FMD virus injected into the tongue. Animals are observed for foot lesions. From these data, the fraction of the standard dose of vaccine that would protect 50% of exposed cattle is then estimated. The reciprocal of this is the PD_50_ value [5,16]. This is a measure of vaccine potency, reflecting protective efficacy.

Owing to concerns about animal welfare, cost and laboratory pathogen escape, the number of animals used for challenge evaluation is generally small and the length of follow-up limited. Consequently, results can be statistically uncertain.

Challenge studies allow a high level of control over characteristics of the participants and pathogen exposure, minimizing differences between vaccinated and control groups. Accurate and detailed outcome measures can improve the statistical power when sample size is small.

Challenge studies provide a standardized platform on which to compare different vaccines for the same disease or to compare the effect of specific variables on vaccine protection. However, the challenge may not mimic natural pathogen exposure and under field conditions many factors will vary in ways which are not captured.

### (b) Randomized controlled trials

This approach is used more routinely in the evaluation of human vaccines. In a randomized controlled trial (RCT), a study group that represents the population of interest is identified, preferably with a high incidence of the disease. Individuals within this population are then selected at random to be vaccinated, or to receive either no vaccine, a placebo or an alternative vaccine. This latter point is important as people may act differently if they think they have been vaccinated. Vaccine storage and delivery is done exactly according to the manufacturer's instructions. The protective efficacy of the vaccine can then be calculated by comparing the incidence in the vaccinated and control groups (equation (1.1), table 1).

RCTs are often referred to as the ‘gold standard’ for assessing the effect of public health interventions [20]; one reason being that vaccinated and control groups have similar levels of exposure to all known and unknown confounding risk factors owing to the randomization process.

As well as being used for pre-licensure phase III trials (see the electronic supplementary material, table S1), national health agencies may perform RCTs to evaluate the likely efficacy of a particular vaccine schedule. Sometimes trials are performed under programmatic conditions to obtain estimates of field protection, called vaccine effectiveness rather than efficacy, the latter being measured under ideal conditions.

The European Medicines Agency (EMA) specifies guidelines and standards for RCT designs for veterinary vaccines [21]. Although field trials are used for veterinary vaccines, unlike human medicine, they are sometimes thought of as inferior methods of efficacy evaluation compared to the standardized and highly controlled conditions of the challenge study [5]. In addition, the cost associated with large trials poses a problem for some veterinary vaccines for which the market is relatively small [2,4]. Furthermore, as entire groups of livestock are typically vaccinated at the same time, cluster randomized designs (considered later) may be more relevant than trials, where vaccinated and unvaccinated individuals exist in the same herd.

### (c) Vaccine effectiveness evaluation: observational studies

Observational studies are the main method of evaluating human vaccines once used in the population at large [12,22–24]. This approach has been neglected in animal populations, although there are some examples of its use [25,26].

Several different observational study designs exist (some key designs are described later). Most calculate the vaccine effectiveness statistic based on the standard formula (equation (1.1), table 1) [6]. For these studies, the term vaccine effectiveness is used, denoting that the evaluation is of vaccine performance under programmatic conditions where vaccine storage, delivery and participant health status will vary.

In an RCT, a vaccine is administered to individuals chosen at random. This is not the case for observational studies, where vaccinated individuals are likely to differ from those not vaccinated in ways that may confound the vaccine effect.

#### (i) Cohort studies

In a cohort study, incidence (risk or rate) is compared in vaccinated and unvaccinated groups over the period of observation. Controlling for differing levels of pathogen exposure is vital and sometimes challenging. In some cohort studies, only individuals from affected subgroups or households are included (household secondary attack rate study); the assumption is made that individuals living in the same house as a case receive a similar pathogen exposure [24].

Compared to a prospective study, conducting a study retrospectively increases the chance of obtaining incorrect data as the passing of time and outcome status may affect recall.

Cohort studies are often used to evaluate human vaccines, sometimes as part of large, ongoing studies [27] or during opportunistic, retrospective analysis of an outbreak [28]. Where national databases with health records for all individuals exist they can be used for national studies of vaccine effectiveness [29]. Large cohort studies are less common for livestock, partly because of cost. Retrospective studies, using either farm records or after outbreaks among small-holders, are more feasible [26].

#### (ii) Case–control studies

It is also possible to estimate vaccine effectiveness by comparing prior vaccination status of affected individuals with the vaccination status of controls that were similarly exposed, but failed to contract the disease [30]. Vaccine history is collected retrospectively and confounders must be adjusted for.

This is a common method of human vaccine effectiveness evaluation. As it is relatively quick and inexpensive to perform [31], the method would be suitable for veterinary vaccines provided that accurate vaccination and disease data are available. However, the lack of vaccinated and unvaccinated animals on the same premises and increased likelihood of vaccination in high-risk groups may prevent identification of a suitable control group. The method may also not be possible within a highly effective control programme owing to the lack of cases.

#### (iii) Vaccine programme impact

A change in vaccination strategy may be assessed in a vaccine impact study by comparing disease burden within a population or cohort before and after the change [32]. Potential bias from underlying temporal trends must be considered; also such studies require good pre- and post-vaccination disease surveillance to accurately detect changes in disease burden. A fall in incidence could be because of vaccine effect or some other factor. If incidence does not fall, the programme is not achieving its objectives either due to low vaccine coverage or effectiveness, although increases in other drivers of disease may have coincided with vaccination.

This problem can be overcome to some extent if vaccine implementation is phased in over time rather than all at once, allowing contemporaneous comparison of vaccinated and control populations. Confounding is further controlled in a ‘stepped wedge design’, where the vaccine is introduced in several steps. By randomly selecting which regions are included in each step, vaccinated and yet to be vaccinated regions are balanced in terms of confounders (see Cluster randomized trials section) [33].

Changes in incidence are routinely assessed in both human and veterinary vaccination programmes, sometimes correlating incidence with coverage. Sero-prevalence surveys are often used for livestock as an unbiased measure of disease burden where under-reporting is a problem. However, sero-positivity owing to infection must be distinguishable from vaccine-induced sero-positivity.

The burden of disease prevented by a human vaccine is sometimes estimated as a function of vaccine coverage, vaccine effectiveness and pre-vaccination disease incidence.

#### (iv) Relative effectiveness

The level of protection afforded by a vaccine can be compared to that of another vaccine or a different schedule to give an estimate of *relative effectiveness*. Many of the above studies (including RCT) can be adapted for this situation.

#### (v) Outbreak studies

Many of these study designs are based on observations made during outbreaks, often through retrospective analysis. When there is a lack of unvaccinated animals, inadequate protection may be identified by outbreaks in vaccinated populations without comparison to a control group. However, it may be difficult to quantify the level of vaccine effectiveness. This may be the case when evaluating outbreaks in commercial farms with uniform management.

Evaluation of reactive vaccination performed in response to outbreaks can be challenging as the investigator may be unsure if individuals were already immune before vaccination, challenge may occur before vaccinated individuals have responded to the vaccine and those left unvaccinated may have a different risk of pathogen exposure.

### (d) Serological evaluation

#### (i) Correlates of protection

Vaccines often induce a measurable response (e.g. antibody titre). If this response is correlated with protection against disease or infection it can be used as an alternative outcome for vaccine evaluation [1,9,10]. Correlates of protection are widely used for both human and veterinary vaccines [34].

In recent times, certain human vaccines, notably meningococcus C in the UK [35] and meningitis A vaccine in Africa [36], have been licensed based on serological correlates of protection without a stage III RCT with the proviso that close monitoring of vaccine effectiveness is performed after introduction of the vaccine. There is pressure to minimize the use of animal challenge studies [37], evaluating serological measures of protection instead [38] (box 2). Although serological studies are routinely used in the evaluation of veterinary vaccines, sero-conversion *per se* is rarely used as a measure of efficacy during licensure.

Box 2. **Correlates of protection—expected percentage of protection (EPP).**

The EPP is a standardized test used to assess the potency of FMD vaccines using serology rather than pathogen challenge. In this method, the sera from 16 to 30 cattle between 18 and 24 months of age, taken 30 days post-vaccination are assessed for their ability to neutralize or bind virus (typically the vaccine strain) using a virus neutralization (VN) test or an ELISA. The proportion of animals expected to be protected is then estimated by referring to serological titres and observed protection from multiple previous challenge studies [5,39].

Many traditional livestock systems do not keep written or computer records. Although some modern commercial farms keep excellent individual animal production records, many do not. This is due to the large number of individuals kept on one farm, the limited value of individual animals and the high rates of population turnover and movements. As sero-status can act as a record of prior infection or present immunity, it is widely used in veterinary settings. However, even if vaccination status is known, with a single serum sample it may be impossible to tell whether infection came before or after vaccination limiting its use for efficacy estimation.

#### (ii) Post-vaccination sero-conversion surveys

In human vaccination campaigns, sero-conversion studies are sometimes performed using pre- and post-vaccination sera to assess vaccine response [23]. This is typically used for phase II immunogenicity trials.

Similar surveys using only sera collected post-vaccination are common in livestock. The proportion with an antibody titre above a specified threshold associated with protection is then determined [40].

#### (iii) Sero-prevalence surveys

This involves assessing sero-status for a representative sample of the population irrespective of vaccination status after a vaccination campaign [23]. Not widely used for human vaccines, these surveys are used in veterinary settings to assess the level of ‘population immunity’ [41], under the assumption that sero-positivity implies protection. Sero-prevalence is a function of the proportion vaccinated, the proportion that sero-convert post-vaccination and the proportion sero-positive following natural infection. In endemic populations, it is therefore difficult to infer if high levels of sero-positivity reflect high coverage with an effective vaccine or widespread infection, or a combination of both vaccination and infection. Where vaccine protection is short-lived or population turnover is rapid, studies need to be regularly updated.

Vaccine effectiveness studies require pathogen exposure and so can only be implemented once outbreaks have occurred, by which time it may be too late to affect the outcome. Assessing population immunity via sero-surveys can detect susceptibility before outbreaks occur; this is useful where vaccination is used in disease-free populations, particularly when reliable estimates of vaccine coverage and effectiveness are not available. This method has proved useful for veterinary vaccines and could aid the evaluation of human population immunity when vaccine records are poor and a measureable correlate of protection exists.

#### (iv) *In vitro* vaccine matching assays

The likely performance of vaccines may sometimes be predicted via *in vitro* serological methods. Antigenic match between influenza vaccines and field viruses is assessed using sera from vaccinated ferrets, or sometimes people, measuring the sera's ability to react with the field viruses [42]. However, these matching studies do not consistently predict effectiveness; currently, the same is true for alternative predictors of match based on genetics [43]. A similar veterinary assay is the ‘r-value’ [39] (box 3).

Box 3. ***r*-value test.**

The ‘*r*-value’ is an *in vitro* assay of FMD vaccine match; this is a measure of the relative reactivity of sera from vaccinated cattle to the field virus in question compared to the reactivity of the same sera to the virus strain used to make the vaccine, performed by ELISA or VN [5,39].

For FMD, a suboptimal vaccine match may be compensated for by having a more potent vaccine that stimulates greater antibody production, e.g. one that contains more antigen per dose [39,44]. The test provides rapid results, but there can be problems with test repeatability [39] and results do not tell you whether the vaccine is actually protecting animals in the field.

#### (v) Cell-mediated correlates of immunity

Although most correlates of protection measure the humoral immune response, cell-mediated immune response is increasingly assessed in human vaccine studies. This is primarily for intracellular infections such as tuberculosis [45]. Assays typically assess the antigen-specific response of T cells (e.g. ELISpot) and their associated cytokines, such as IFN-γ, used to evaluate BCG vaccination in humans [46], cattle [47] and badgers [48].

Combining information on vaccine potency and antigenic match improves the prediction of efficacy [44] with identification of genetic predictors under development [49].

### (e) Direct versus indirect effects of vaccination

Direct vaccine protection is the reduction in risk in vaccinated compared with *similarly exposed* unvaccinated individuals. However, vaccinating some but not all members of a group can result not only in protection of those vaccinated, but also reduced pathogen exposure and morbidity in those not vaccinated. This indirect vaccine effect is due to a reduction in transmission within the group as a whole. Studies that only capture the direct effect of vaccination by comparing vaccinated and unvaccinated individuals in the same group may underestimate the overall effect of vaccination by not capturing the indirect effects.

#### (i) Cluster randomized trials

**Figure 1.**
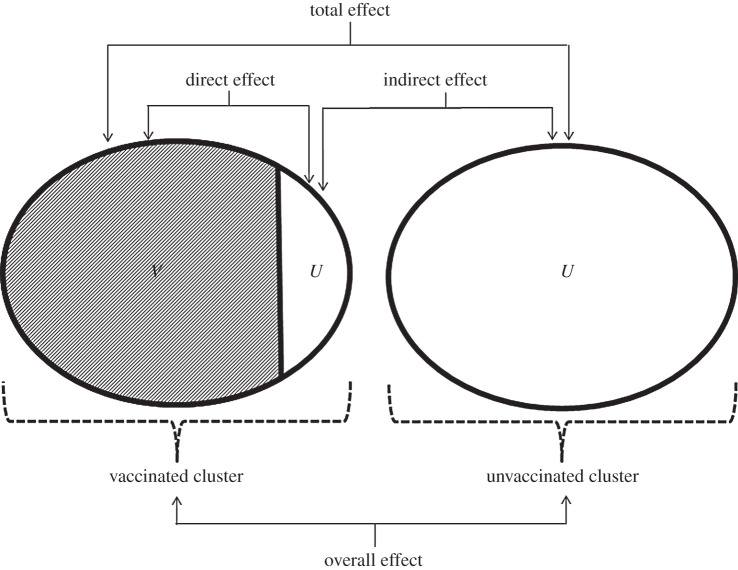
Diagram showing the different types of vaccine effect detectable in a cluster trial and which vaccine groups to compare to estimate them. Within a cluster, *V* and *U* represent vaccinated and unvaccinated individuals, respectively [6]*.* Using this design, the different effects (direct, indirect, total and overall) can be estimated by comparing groups as indicated by the arrows. Coverage in the vaccinated cluster is <100%.

In cluster randomized trials (CRTs), the intervention is randomly allocated to entire clusters, rather than individuals. Certain CRTs (and observational vaccine effectiveness studies) can be designed so as to capture direct and indirect vaccine effects (figure 1).

By randomizing allocation to different clusters, rather than individuals within the same cluster, inferences can be made on the overall effect of vaccination on a community, rather just the direct effects afforded to the individual [13,22]. Vaccinated and control clusters will tend to be similar due to randomization.

CRTs are increasingly seen as the most relevant study design for informing policy and are widely used to evaluate human vaccines. The group management of production animals naturally lends itself to this study design and, although the term CRT is rarely used, the method is common, especially for fish and poultry [50,51]. However, CRTs have seldom if ever been conducted for veterinary vaccines used in national control programmes of notifiable diseases such as FMD, brucellosis or peste des petits ruminants.

## 3. Vaccine coverage

As well as evaluating whether vaccinated individuals are protected, it is crucial to check that a sufficient proportion of individuals receive the vaccine as per the vaccine schedule (i.e. vaccine coverage). There are several ways of assessing coverage (see below) [12]. Although the most costly often provide the best data, they may not always be necessary.

- Distributed method: the simplest approach is to determine the number of doses distributed divided by the target population size. But this does not reveal if individuals received the full course or account for wasted/unused doses. Inaccurate estimates of target population size will bias coverage estimates.
- Administered method: examining the number of doses actually administered from central or local records can provide more accurate and detailed measures of coverage.
- Surveys: surveys may be based on convenience samples (e.g. schools), though the population sampled may not be representative of the population at risk. Alternatively, structured surveys can be implemented, for example the WHO 30 cluster, two-stage stratified random survey [52].
- Sero-prevalence: finally, coverage may be partially inferred from sero-prevalence surveys (see above), but difficulties distinguishing prior infection from vaccination may exist.

The recommended method of coverage monitoring will vary depending on the setting, for example, in areas with only well-organized commercial farms it may be satisfactory to use routinely recorded data to monitor coverage at the herd level assuming that vaccination is then applied to all eligible animals within a herd. In other settings, this assumption will be incorrect and routinely recorded data may not be reliable.

## 4. Discussion

Under field conditions, the performance of both human and veterinary vaccines can vary unexpectedly. There are various reasons for this, including vaccine factors, such as variable batch potency, poor administration, failure to observe shelf-life and cold chain requirements; pathogen factors such as level of challenge and the emergence of novel field strains with poor vaccine match; and host and environmental factors that influence immune response, such as genetics and nutrition. Furthermore, population density and nature and frequency of contacts will influence level of challenge. Often variation in protection cannot easily be explained, let alone predicted (e.g. [30,53]). Without ongoing vaccine evaluation, including monitoring of effectiveness and coverage, it will be difficult to anticipate and explain breakdowns in disease control within a vaccination programme.

In the field, a vaccine will have to protect individuals of differing susceptibility and pathogen exposure level. Human and veterinary medicine deal with this in very different ways. Field trials evaluating human vaccines are designed to include much of this variation. If a vaccine is to be used in a setting different to previous trials, further studies may be conducted. The protective effect of a veterinary vaccine is often assessed in small studies which minimize variation in animal susceptibility and exposure level. In theory, veterinary vaccines are then formulated with a potency that is expected to protect even when animal susceptibility and pathogen exposure are high [34]. Despite this, vaccine failure in the field can still occur.

The authorization process is fundamentally different for human and veterinary vaccines. Human vaccine licensing is based on limited controlled laboratory studies and extensive clinical trials and field effectiveness studies. Veterinary vaccines are authorized on the basis of more extensive controlled laboratory studies involving pathogen challenge, backed up (usually) by field studies which are less extensive. Human health is largely overseen by public bodies with funds available for large field trials, whereas animal health is largely dealt with by the private sector which has a limited capacity to fund and coordinate extensive vaccine evaluation studies. Unlike for key human diseases, incidence, prevalence and antigenic change are rarely monitored systematically to inform veterinary vaccination policy.

As governmental interest in livestock health is largely limited to notifiable diseases, most countries lack coordinated control programmes for endemic, non-notifiable diseases that cause ongoing losses to the livestock sector. By contrast, ministries of health try to limit the impact of all human diseases, both endemic and exotic. As disease control on one farm affects the disease risk faced by others, central coordination is required for a programme to be effective. In developed countries, governmental veterinary vaccination programmes typically concern the short-term control of outbreaks of exotic or emerging pathogens (e.g. FMD, bluetongue). For endemic veterinary diseases, vaccination may be applied routinely, often with inconsistent evaluation and limited ability to adapt to the situation on the ground; this is particularly true where vaccination is left to the private sector. By contrast, the enormous reduction in vaccine preventable childhood diseases, seen over the past 50 years, would not have occurred without central coordination despite the enormous interest parents have in the health of their children.

### (a) Possibilities for veterinary vaccine effectiveness evaluation

Despite its importance, the area of effectiveness evaluation has been little explored for veterinary vaccines. The under-utilization of vaccine effectiveness studies in the veterinary sector becomes even more apparent when one considers the ever-increasing pressure to reduce the use of animals in experimental studies. So far in the veterinary field, this has largely been addressed through the use of correlates of protection without supporting evidence from studies of protection in the field.

Organizations involved in drug authorization, including the EMA, are currently considering whether pharmaceuticals, including vaccines, could be licensed using data for a limited range of indications with the possibility of adding further indications later on using post-authorization studies. However, in the veterinary field, this has been hampered by the lack of established methodology for vaccine effectiveness evaluation. To assist researchers interested in this area, recommended methods for doing this have been described in the electronic supplementary material.

## 5. Conclusion

The importance of independent vaccine evaluation including quality assurance cannot be emphasized enough. Both vaccine evaluation and quality assurance were crucial for the global eradication of smallpox and rinderpest. The cost of thorough evaluation can be justified when one looks at the huge overall cost of vaccination programmes, the uncertainty that often exists about effectiveness and the major benefits experienced when programmes are successful.

In order to evaluate vaccine programmes in the field, a number of challenges need to be addressed. These include the paucity of individual disease and vaccine records and difficulties in finding appropriate vaccinated and unvaccinated comparison groups. When suitable records are not available but a good correlate of protection is known, sero-surveys have provided useful estimates of livestock population immunity. This evaluation method may be appropriate for human vaccines when effectiveness studies are not possible.

Vaccine effectiveness studies are essential for measuring protection actually achieved within a vaccination programme. Cluster trials performed under ordinary field conditions are particularly informative [22]. Adoption of these methods by the veterinary sector would provide a better understanding of the full benefits and costs of vaccination. This evidence base would help to secure funding for effective disease control and leave less room for speculative policy-making. However, evaluation of protection at the population level requires central coordination.
